# Review of the Mechanical Properties and Numerical Simulation of Composite Solid Propellants

**DOI:** 10.3390/ma16216875

**Published:** 2023-10-26

**Authors:** Jianru Wang, Peng Cao, Xiaoxu Wang

**Affiliations:** 1 The 41st Institute of the Fourth Research Academy of CASC, Xi’an 710025, China; wjr104zah@sina.com; 2 Faculty of Architecture, Civil and Transportation Engineering, Beijing University of Technology, Beijing 100124, China; x1059601389@163.com

**Keywords:** composite solid propellant, mechanical response behavior, macroscopic constitutive model, mesoscopic mechanical model, microscopic molecular model

## Abstract

Investigating the constitutive relationship and the damage failure mechanism of solid propellants is of significance for improving the safety, storage period and use efficiency of solid rocket motors. This paper focuses on the complex mechanical response behavior of composite solid propellants under loads and introduces experimental research on quasi-static and dynamic mechanical properties. Limited by the accuracy of instruments and testing methods, the research progress of macroscopic constitutive models, mesoscopic mechanical models and microscopic molecular models is summarized from the perspective of numerical simulations based on model scale and modeling methods. This paper tracks the historical progress of key models and summarizes the main achievements and prospects in this field. The research in this paper has high scientific and theoretical significance and engineering application value. It can provide an important reference and guidance for the structural optimization and performance improvement of solid propellants and lay a solid foundation for the development of solid rocket motors.

## 1. Introduction

**Figure 1:**
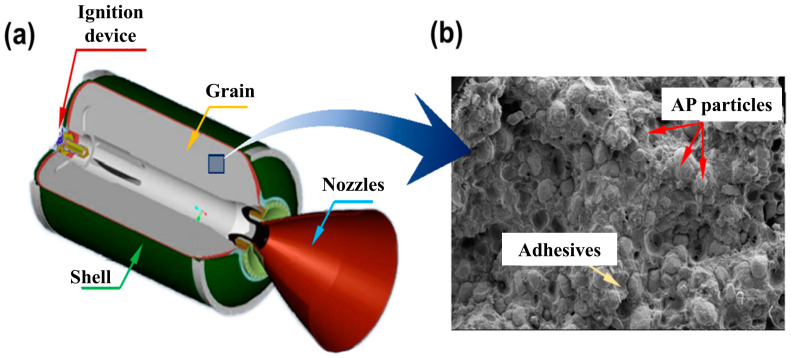
(**a**) Schematic diagram of solid rocket motor structure composition [4]; (**b**) microstructure of composite solid propellant [4].

With the development of aerospace technology, solid rocket motors have been widely used in aerospace, carrier rocket, military weapon and other applications due to their advantages of simple structure, high safety, good performance, small volume, long storage cycle and convenient use [1,2]. A solid rocket motor includes a composite solid propellant, shell, ignition device and stabilizing device, as shown in Figure 1. Solid rocket motors are subjected to various loads such as thermal radiation, impact, vibration and pressure during manufacture, transportation, ignition and flight, which may lead to structural damage and affect the normal operation of solid rocket motors. Composite solid propellants occupy a crucial part in the design of solid rocket motors, ensure the structural integrity of the composite solid propellant and affect the safety and reliability of the entire system. Therefore, mastering the mechanical response behavior and constitutive relationship of a composite solid propellant under load will help guide and design the solid rocket motor, save resources and improve service efficiency [3].

A composite solid propellant is energetic material that is composed of a polymer binder as a matrix, an oxidizer and metal fuel as a doping phase. The mechanical properties of composite solid propellants are determined by the material properties and structure of each component. To reasonably design structural components and avoid resource waste, it is necessary to investigate the stress–strain relationship of structural components, which plays an irreplaceable role in the performance optimization of composite solid propellants [5,6,7]. The deformation and evolution of the internal structure of a composite solid propellant under stress is complicated, and there are many difficulties in conventional experimental research methods. Therefore, a numerical simulation based on a refined analysis model becomes an effective research method. There are three main ways to investigate the mechanical properties of composite solid propellant by numerical simulation [8]: ignoring microstructure, simplifying to homogeneous material, and simulating constitutive relationship; establishing a mesoscopic model, considering the relationship between microstructure and macroscopic mechanics; investigating the damage evolution mechanism at the molecular and atomic scales.

In this paper, the research progress and development status of mechanical property characterization of composite solid propellants are introduced. Limited by the accuracy of instruments and testing methods, the numerical simulations including the macroscopic constitutive model, mesoscopic mechanical model and microscopic molecular model are summarized based on model scale and modeling methods, and their features and weaknesses are presented in Table 1. According to the main results of the review, the future development trend of composite solid propellants is proposed, important theoretical guidance for the structure optimization and performance improvement of composite solid propellants is provided, and a solid foundation is laid for the development of solid rocket motors.

## 2. Mechanical Property Characterization of Composite Solid Propellants

The mechanical properties of composite solid propellants can be characterized by quasi-static and dynamic mechanical property experiments according to the range of strain rate. Composite solid propellants have a high binder content and large deformation under load and exhibit typical viscoelasticity. The formula, material properties and loading conditions (temperature and strain rate) of a composite solid propellant all affect the stress–strain curve.

### 2.1. Quasi-Static Mechanical Properties

**Figure 2:**
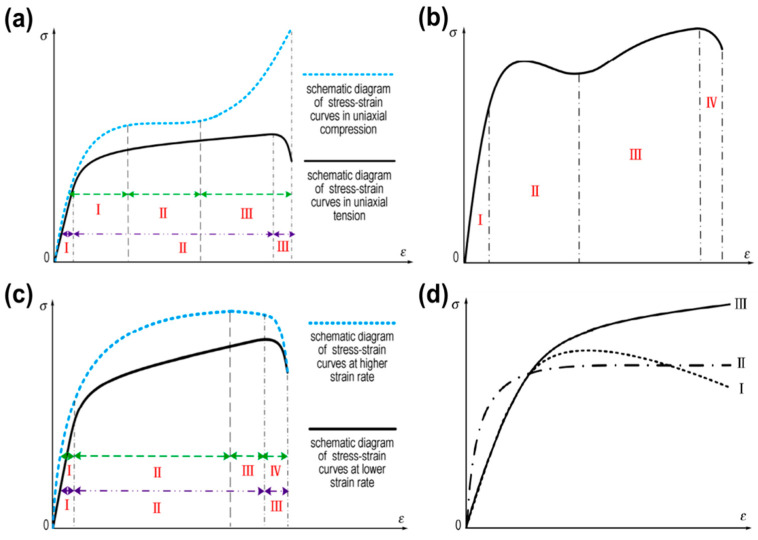
Stress–strain curves of composite solid propellants under different loading conditions: (**a**) uniaxial compression (I—linear elastic section, II—stress-hardening section, III—failure instability section) and uniaxial tension (I—linear elastic section, II—“dewetting“ damage section, III—failure section); (**b**) low-temperature quasi-static compression (I—initial linear section, II—yield and strain-softening section, III—strain-strengthening section, IV—failure section); (**c**) high-strain-rate compression (I—initial linear section, II—stress-hardening section, III—strain-softening section, IV—failure section) and low-strain-rate compression (I—linear elastic section, II—stress-hardening section, III—failure instability section); (**d**) low-temperature quasi-static uniaxial tension (I, II—yield factor dominate, “dewetting“, poor interface bonding, curve shape showing tail dropping or plateau; III—deformation factor dominate, good interface bonding, curve shape showing upward) [17].

Under quasi-static conditions, the tensile stress–strain curve of a composite solid propellant presents three typical stages: linear elastic section, “dewetting“ damage section and failure section, while the compressive stress–strain curve presents a linear elastic section, a stress-hardening section and a failure instability section, as depicted in Figure 2a. Francis and Carlton [9] believed that the “dewetting” damage section indicated the propagation of microcracks in the composite solid propellant, leading to the separation of particles from the matrix, a reduction in bearing capacity and a nonlinear stress–strain curve. Lai et al. [10] believed that the stress-hardening section was the plastic deformation caused by macroscopic cracks inside the composite solid propellant.

Figure 2b shows that the stress–strain curve of a composite solid propellant presents multiple stages, namely an initial linear section, a yield and strain-softening section, a strain-strengthening section and a failure section [11,12], at low temperature. Researchers [13,14,15] believed that the temperature decreased, the binder crystallized, resulting in multiple stages on the stress–strain curve of the composite solid propellant, and the elongation at break increased. Landsem et al. [11] believed that the particles inside the composite solid propellant reduced the bond strength, and “dewetting” occurred during small strain and reduced the stress value, resulting in the appearance of a strain-softening section. Hu et al. [12] believed that under the quasi-static high strain rate, the composite solid propellant produced plastic deformation, and the internal heat could not dissipate, resulting in the decrease in stress and the appearance of a strain-softening section. Yang et al. [16] used the fuzzy subgroup theory to analyze the stress–strain curve of a composite solid propellant, and the curve of the composite solid propellant was determined by yield factors and deformation-hindering factors, as shown in Figure 2d.

**Figure 3:**
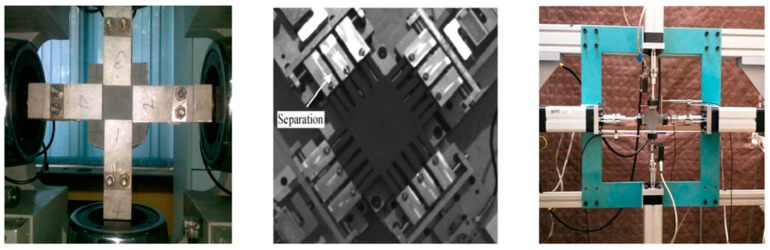
Solid propellant cross biaxial tensile experiments [17,21,22,23].

The biaxial mechanical property testing methods of composite solid propellants include the slat test [18,19,20] and cross test [17,21,22,23]. The biaxial tensile test of slats is limited to the range of elastic deformation with small strain [19], and the experimental device is depicted in Figure 3. Zhang [21] found that a composite solid propellant had different properties of cross-section samples in all directions through the biaxial mechanical property experiments. Researchers [17,21,22,23] found that a composite solid propellant had an obvious bidirectional weakening effect under biaxial tensile testing.

The mechanical property testing methods for composite solid propellants under a quasi-static triaxial state include the “poker chip” experiment using a round thin slice [24], the radial compression experiment using a thick disc [25], the pipe fitting experiment [26] and the confining pressure experiment [27]. The latter two test methods can well simulate the mechanical properties of a composite solid propellant under an ignition and pressurization environment. Kumar [26] found short relaxation time had no effect on the relaxation modulus of a composite solid propellant through quasi-static relaxation experiments. With the increase in relaxation time, the relaxation modulus of the pipe increased by 18~30% in the slat tensile test and 25~43% in the uniaxial tensile test. Liu et al. [27] analyzed the stress state of a composite solid propellant under confining pressure, and the results showed that high triaxial tensile stress was present on the surface of particles. The tensile stress surrounded the triaxial tensile stress under ambient pressure, while the compressive stress surrounded the triaxial tensile stress under external pressure, which inhibited the initiation and evolution of damage, and the material had a high strength and modulus. Zhang [28] found that the existence of confining pressure was conducive to the long-term storage of composite solid propellants. Based on confining pressure experiments, Zhang et al. [29] analyzed the mechanical properties of a composite solid propellant under triaxial stress and established corresponding strength criteria.

Liu [30,31,32,33,34] investigated the fracture mechanical properties of a composite solid propellant under quasi-static tensile conditions using an intermediate penetrating plate crack test piece. The effects of temperature, strain rate, thickness, crack width, stress state and aging time on crack growth were determined, and a crack growth model was established.

### 2.2. Dynamic Mechanical Properties

Currently, the separated Hopkinson pressure bar (SHPB) technique is used to characterize the dynamic mechanical properties of materials at high strain rates. Sun et al. [35] and Balzer et al. [36] added a thermal insulation system to the SHPB device and investigated the dynamic mechanical properties of a composite solid propellant under a high strain rate (>10^2^ s^−1^) and different temperatures. Ho [37] investigated the compression loading of HTPB solid propellant at different temperatures with high strain rate (10^3^~10^4^ s^−1^) and found that the stress–strain curves of HTPB solid propellant all showed three stages: a linear elastic compression section, a ductile deformation stress-hardening section after yield, and a failure and instability section. They considered that there were microcracks and holes in the solid propellant in the linear elastic compression section. In the stress-hardening section, both viscoelastic deformation and viscoplastic deformation occurred, cracks propagated and secondary damage existed. Lai’s results from ref. [17] conducted uniaxial compression experiments on HTPB solid propellant at low temperature and high strain rate (7 × 10^2^~2 × 10^3^ s^−1^), and found that the stress–strain curve presented three-stage characteristics consistent with quasi-static conditions at low strain rate; at high strain rate, it presented multi-stage characteristics, which were related to the cracking of solid particles inside the propellant. Wang et al. [17] conducted dynamic uniaxial tensile experiments on HTPB solid propellant in the strain rate range of 1~10^2^ s^−1^ and found that at normal temperature, the stress–strain curve of the solid propellant presented three-stage changes, and at low temperature and higher strain rate, the multi-stage characteristics were related to the solid particle cracking, and the glass transition temperature changed inside the propellant. With the decrease in temperature and the increase in strain rate, the multi-stage characteristics became more obvious, as depicted in Figure 2b.

**Figure 4:**
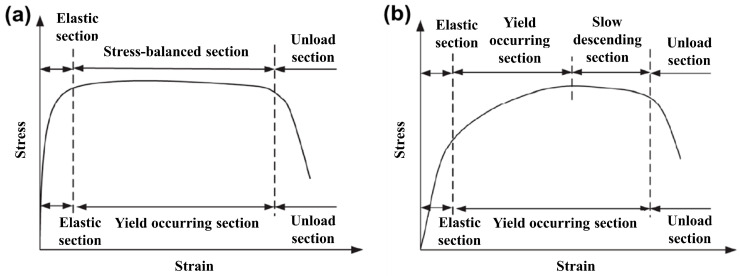
Stress–strain curves of solid propellants with (**a**) small particle size and (**b**) large particle size [40,41].

Cady et al. [38] investigated the dynamic mechanical properties of HTPB solid propellant at a high strain rate, combining the sample size, stress balance, initial elastic modulus, stress yield, glass transition and other factors. Jennifer et al. [39] found that with the increase in plasticizer content, the strength of HTPB solid propellant and the glass transition temperature also decreased. Field et al. [40] studied the effects of four different particle sizes (3–300 μm) of ammonium perchlorate (AP) particles on the dynamic mechanical properties of hydroxy-terminated polybutadiene (HTPB) solid propellant and found that the strain rate correlation was not obvious at room temperature, while the strain rate correlation was significant at low temperature. The stress–strain curve of a solid propellant with a small particle size had obvious stress “plateau region” [40,41] and presented three stages: linear elastic section, constant stress section and failure section, as depicted in Figure 4a. The smaller the particle size of solid particles, the more difficult “dewetting”, the larger the total active surface, the more “additional crosslinking points” and the stronger the overall role of resistance to external forces, which was manifested as a stress platform on the macro level. Figure 4b shows the stress–strain curve of a solid propellant with a larger particle size, showing the variation law of ascending stage, slow ascending stage–slow descending stage–unloading stage. The larger the solid particles, the easier to “dewetting”, the smaller the relative specific surface area, the smaller the total active surface, the fewer “additional crosslinking points” and the weaker the resistance to external forces, resulting in microcracks, and holes and other defects, gradual intensification and eventual failure.

The fracture mechanical properties of composite solid propellants under an ultra-high strain rate are investigated based on flight-impact experiments. The Stanford Research Institute (SRI) investigated the fracture behavior of a composite solid propellant using a flight-impact experiment and established a fracture model of a composite solid propellant based on fracture dynamics [42]. Huang et al. [43] analyzed the stratification phenomenon after the fracture of a composite solid propellant based on flight-impact experiments. Long et al. [44,45] constructed the main curve of the fracture strength factor using the central straight crack disc test, which provided a new idea for the fracture mechanical properties of a solid propellant at a high strain rate.

The mechanical behavior of a composite solid propellant is complicated, and there are still many problems. It is quite difficult to investigate the mechanical properties of composite solid propellants under multiaxial stress. The experimental data are relatively discrete, and the experimental studies on biaxial compression and biaxial tension–compression are few. The experimental studies on the mechanical properties of composite solid propellants under dynamic loading conditions are not comprehensive, and there is a lack of experimental studies under higher strain rate conditions, and there are few experimental studies on dynamic tension and compression under multiaxial stress conditions. Therefore, due to the complexity of experimental research on the mechanical properties of composite solid propellants, there is an urgent need to develop numerical simulation methods to further and comprehensively investigate the mechanical behavior of composite solid propellants.

A composite solid propellant is a typical viscoelastic material, and it is sensitive to time and temperature, so its constitutive model is complicated. The constitutive model of composite solid propellants can be studied from the following three aspects: based on macroscopic mechanics, combine elastic elements and sticky pot elements to form viscoelastic properties, and establish a viscoelastic constitutive model by introducing viscoelastic functions or parameters; based on mesomechanics, establish a mesoscopic constitutive model by considering the stress concentration, microcracks and micropores in the material during loading; at the micro- and nanoscopic levels, establish the force field model of molecules to investigate the interaction and structure evolution of each component of a composite solid propellant. This paper introduces the characteristics, application scope, differences and relations of the three models of composite solid propellants and forecasts the future development trend.

## 3. Macroscopic Constitutive Model of Composite Solid Propellant

A composite solid propellant is a composite material with a polymer binder as a matrix, is filled with a large number of solid particles and shows typical nonlinear viscoelasticity. Based on the Boltzmann superposition principle, Leaderman [46] introduced a nonlinear stress function and transformed the viscoelastic constitutive model of an integral line into a nonlinear viscoelastic constitutive model:

(1) $$\sigma (t)=E_{0}h_{0}\left[\varepsilon (t)\right]+\int_{0}^{t}\Delta E\left(\psi^{t}-\psi^{\tau }\right)\frac{dh_{1}\left[\varepsilon (\tau )\right]}{d\tau }d\tau$$

where $h_{0}$ and $h_{1}$ are nonlinear functions of strain and ψ is the reduction time. This model was suitable for low strain rates and small deformation but did not consider thermodynamic factors.

Based on irreversible thermodynamics, Schapery [47,48] introduced Gibbs free energy and established a relaxed nonlinear viscoelastic constitutive model with reduction time:

(2) $$\sigma (t)=E_{\theta }h_{\theta }\left[\varepsilon (t)\right]+h_{1}\int_{0}^{t}\Delta E\left(\psi^{t}-\psi^{\tau }\right)\frac{dh_{2}\left[\varepsilon (\tau )\right]}{d\tau }d\tau$$

where $h_{e},{\;h}_{1}\;and\;h_{2}$ are nonlinear functions of strain. This model embodied the time–temperature equivalent principle and described the nonlinear mechanical properties of a composite solid propellant under a low strain rate and small deformation [49,50,51]. Based on Schapery’s constitutive model, some researchers [52,53,54] introduced a damage function to construct an integral nonlinear viscoelastic constitutive model of a composite solid propellant containing damage under quasi-static loading, reflecting the evolution of damage with the change in the macroscopic elastic modulus. Tscharnuter et al. [55] introduced the Perzyna viscoplastic model on the basis of the Schapery model to establish a viscoelastic–plastic constitutive relation. Zhang [56] extended the nonlinear viscoelastic–plastic constitutive equation to a three-dimensional form using the incremental method to investigate the viscoelastic mechanical behavior of a composite solid propellant. Wang et al. [57] established the viscoelastic–viscoplastic constitutive model of a composite solid propellant through creep experiments, and they found that the ratio of viscoelastic strain and viscoplastic strain was consistent with the experiment and the viscoplastic strain increased with the increase in stress. Meanwhile, with the increase in loading time, the viscoplastic part had a large gap in the predicted results due to the damage inside the material.

The mechanical response of a composite solid propellant under a high strain rate was investigated using the Zhu–Wang–Tang (ZWT) constitutive model, visco-hyperelastic constitutive model and visual constitutive model. Based on the Green–Rivlin multiple integral theory, the ZWT constitutive model [58] was composed of a nonlinear spring (nonlinear elastic response) and two Maxwell elements (viscoelastic response of quasi-static low strain rate and dynamic high strain rate) in parallel:

(3) $$\begin{array}{l} \sigma (t)=E_{0}\varepsilon (t)+\alpha \varepsilon^{2}(t)+\beta \varepsilon^{3}(t)+\\ E_{1}\int_{0}^{t}\varepsilon (\tau )\exp\left(-\frac{t-\tau }{\theta_{1}}\right)d\tau +E_{2}\int_{0}^{t}\varepsilon (\tau )\exp\left(-\frac{t-\tau }{\theta_{2}}\right)d\tau \end{array}$$

where $E_{0},\alpha \;and\;\beta$ are the elastic coefficients of the nonlinear spring; $E_{1}\;and\;E_{2}$ are the elastic coefficients of the two Maxwell elements, respectively; and $\theta_{1}\;and\;\theta_{2}$ are the relaxation times. The ZWT constitutive model can well describe the effect of strain rate on material nonlinearity, but it can only describe the condition of strain less than 7%. Wang et al. [59] found that the ZWT constitutive model could only describe the mechanical response behavior of the strain within 2% at a low strain rate and within 5% at a high strain rate and could not describe the large deformation of the composite solid propellant.

Pouriayevali et al. [60] connected the hyperelastic element with the viscoelastic element in parallel to construct the viscoelastic hyperelastic constitutive model:

(4) $$\sigma_{11}=\sigma^{e}+\sigma^{v}=\sigma_{11}^{e}+k\int_{0}^{t}\frac{\partial \sigma_{11}^{e}}{\partial \tau }\exp\left(-\frac{t-\tau }{\theta_{}}\right)d\tau$$

where $\sigma^{e}$ is the strain energy function of the superelastic element and $\sigma^{v}$ is the genetic integral of the viscoelastic element. The model was suitable for large deformation and high-strain-rate loading conditions, but the effect of temperature load was not considered. Yildinm and Oezupek [61] introduced the Yeoh strain energy function to describe the visco-superelastic constitutive model of a composite solid propellant with an aging factor at a high strain rate. Chang et al. [62] adopted the Moonev–Rivlin strain energy function to improve Burke’s constitutive model, and constructed a viscosity–hyperelasticity constitutive model of HTPB solid propellant deformation with a temperature factor at a high strain rate.

Ho [37] used the strain energy function to describe the damage evolution in the deformation process of composite solid propellants, and constructed a phenomenological nonlinear viscoelastic constitutive model of HTPB solid propellant at a high strain rate. However, the strain energy function needs to be constructed piecewise; therefore, it cannot describe the deformation of a composite solid propellant in the range of low temperature and large strain rate.

For the macroscopic constitutive model of composite solid propellants, there are some deficiencies: the existing nonlinear viscoelastic constitutive model cannot describe the multi-stage characteristics and deformation failure process of the stress–strain curve of composite solid propellants; it is difficult to determine the damage function; the ZWT constitutive model and visco-hyperelastic constitutive model cannot accurately describe the mechanical behavior of a composite solid propellant under large strain and a high strain rate. The mechanical properties of composite solid propellants are closely related to the properties, structure, distribution, content and interfacial properties of each component. Therefore, it is necessary to establish a granular damage mechanical model on the mesoscopic level to establish the influence law of the mesoscopic structure and mechanical properties and accurately describe the mechanical response behavior and failure process of a composite solid propellant.

## 4. Mesoscopic Mechanical Model of Composite Solid Propellant

A composite solid propellant is a composite material with multiple components and a high particle content. There is an interface between components. Under load, the interface between components is debonded, and the particles undergo “dewetting“, affecting the mechanical properties.

### 4.1. Mesoscopic Constitutive Model

**Figure 5:**
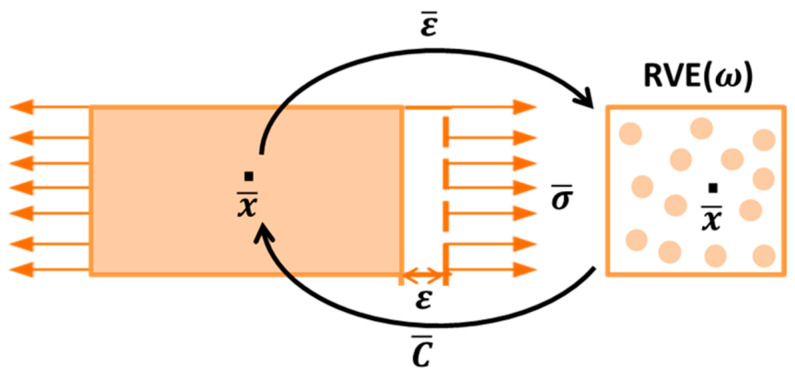
Schematic diagram of mesoscopic mechanical model.

To investigate the mechanical properties of composite solid propellants at the mesoscopic level, it is necessary to establish a real particle-filling model. To simplify the numerical simulation, a representative volume element (RVE) is established, and the entire material is treated as a periodic arrangement of the RVE. The RVE contains all the structural properties of the material at the mesoscopic level but is small enough to be considered as a homogeneous point at the macroscopic level. For each macroscopic point $\bar{x}$, given the macroscopic strain $\bar{\varepsilon }$, the macroscopic stress $\bar{\sigma }$ needs to be calculated. At the mesoscopic level, the macroscopic point is regarded as the center of the RVE region ω, and the boundary is ∂ω, as depicted in Figure 5. The Hill–Mandell theory held that the energy on two scales was equal, and the relationship between macroscopic strain $\bar{\varepsilon }$ and macroscopic stress $\bar{\sigma }$ could be converted into the relationship between mean strain $\langle \varepsilon \rangle$ and mean stress $\langle \sigma \rangle$ on the RVE, where $\bar{\varepsilon }=\langle \varepsilon \rangle$, $\bar{\sigma }=\langle \sigma \rangle$. The matrix material has a volume $V_{0}$ and a volume fraction $v_{0}=V_{0}/V$, where V is the volume of the RVE. The total volume of the particle-filled phase is $V_{1}$, and the volume fraction is $v_{1}=V_{1}/V=1-v_{0}$. Under linear boundary conditions, the mean strain of each phase is related to the strain concentration tensor $B^{\varepsilon }$. Based on the Eshelby tensor $S\left(I,C_{0}\right)$, assuming the strain field in the inclusions is uniform and related to the macroscopic strain [63],

(5) $$B^{\varepsilon }={\left\{I+S:\left[{\left(C_{0}\right)}^{-1}:C_{1}-I\right]\right\}}^{-1}$$

where the Eshelby tensor $S\left(I,C_{0}\right)$ depends on the geometry of the particle-filling phase (I) and the stiffness tensor $C_{0}$ of the matrix. Then, the mechanical behavior of a composite solid propellant can be expressed in the following form:

(6) $$\bar{\sigma }=\bar{C}:\bar{\varepsilon },\bar{C}=\left[v_{1}C_{1}:B^{\varepsilon }+(1-v_{1})C_{0}\right]:{\left[v_{1}B^{\varepsilon }+(1-v_{1})I\right]}^{-1}$$

where $\bar{C}$ is the macroscopic stiffness tensor of the composite solid propellant.

The mesoscopic mechanical theory of composite solid propellants is based on inclusion theory, including the Eshelby equivalent inclusion theory [64], self-consistent theory [65,66], Mori–Tanaka method [67] and differential method [68]. Chen et al. [69] combined Eshelby’s equivalent theory and the Mori–Tanaka method to establish the macroscopic constitutive relation of particle-filled composites. Gui et al. [70] established the mathematical relationship between the mechanical properties of NEPE solid propellant and the composition, particle size gradation, binder matrix strength and modulus. Based on Coleman and Noll’s finite viscoelastic theory and polymer theory, Burke [71] believed the nonlinearity of a composite solid propellant was composed of the nonlinear viscoelasticity of the matrix and the linear elasticity of the particles. Using Eshelby’s equivalent inclusion theory, Peng et al. [72] established a linear viscoelastic constitutive equation for composite solid propellants containing spherical and ellipsoidal particles, revealing the strengthening effect of elastic particles on the viscoelastic matrix. In addition to the matrix and particles, there was also a third phase in the composite solid propellant, which was a crosslinked substance formed by the reaction of the binder and hardener. Pang [73] proposed a mechanical model of the transition phase of a composite solid propellant based on the bonding agent interaction model and morphological structure theory:

(7) $$\varepsilon =\frac{v_{f}\sigma_{f}}{E_{f}}+\frac{v_{i}\sigma_{i}}{E_{i}(t)}+\frac{v_{m}\sigma_{m}}{E_{m}(t)}$$

where $\sigma_{f}=\sigma_{i}=\sigma_{m}{;V}_{f},V_{i}\;and\;V_{m}$ are the volume fractions of the particles, transition phase and matrix, respectively.

### 4.2. Interface Cohesion Model

The above research methods on macroscopic and mesoscopic mechanical properties of composite solid propellants simplify the mesoscopic structure, fail to fully consider the characteristics of the mesoscopic structure and cannot describe the details of local fields. They are only applicable to the solution of effective stiffness. However, the equivalent strength is related to many factors such as the particle/matrix interface and mesostructure, and it is difficult to obtain the analytical solution of the equivalent strength through theoretical calculation. The particle/matrix bonding interface is a relatively weak area in composite solid propellant, which is prone to dehumidification and microcracks. Based on Burke’s theory, Peng [74] established a nonlinear viscoelastic constitutive model of a composite solid propellant describing matrix viscoelasticity, particle elasticity and microcracks caused by interfacial dehumidification:

(8) $$\sigma =\int_{0}^{t}\bar{E}(t-\tau )\frac{\partial \varepsilon }{\partial t}d\tau =(1-D)\int_{0}^{t}E_{0}(t-\tau )\frac{\partial \varepsilon }{\partial t}d\tau =(1-D)\int_{0}^{t}\beta E_{m}(t-\tau )\frac{\partial \varepsilon }{\partial t}d\tau \;$$

where $E_{m}$ describes the viscoelasticity of the matrix, β describes the reinforcement effect of particle filling and D is the microcrack damage caused by interface dehumidification. The model reflected the effect of mesocomponent and structural evolution on the macroscopic mechanical properties of the composite solid propellant.

Tan et al. [75] proposed a Mori–Tanaka mesomechanical model with interfacial debonding to describe the nonlinear mechanical behavior of composite solid propellants:

(9) $$\bar{\sigma }=(1-f)\sigma^{m}+f\sigma^{p}$$

(10) $$\bar{\varepsilon }=(1-f)\varepsilon^{m}+f\varepsilon^{p}+f\varepsilon^{\mathrm{int}}$$

(11) $$\bar{\varepsilon }=M^{p}:\bar{\sigma }+f\{(M^{p}-M^{m}):\sigma^{p}+\varepsilon^{\mathrm{int}}\}\;$$

where $M^{p}\;and\;M^{m}$ are the compliance tensors of the particles and matrix, respectively, and $f\varepsilon^{int}$ is the debonding strains of the particles and matrix. Then, they combined the nonlinear bonding rate and micromechanics theory and proposed a nonlinear interface bonding model of a composite solid propellant [76,77,78]. On this basis, Qu et al. [79] replaced the straight-line segment of the stress–strain curve with a parabola and obtained an improved interface debonding model:

(12) $$\left\{\begin{array}{c} \sigma^{\mathrm{int}}=-\frac{k_{\sigma }^{2}}{\sigma_{\max}}{\left(\left[u_{r}\right]-\frac{\sigma_{\max}}{k_{\sigma }}\right)}^{2}+\sigma_{\max}\left[u_{r}\right]≺\frac{\sigma_{\max}}{k_{\sigma }}\;\\ \sigma^{\mathrm{int}}=-\frac{k_{\sigma }^{2}}{\sigma_{\max}}{\left(\left[u_{r}\right]-\frac{\sigma_{\max}}{k_{\sigma }}\right)}^{2}+\sigma_{\max}\frac{\sigma_{\max}}{k_{\sigma }}≺\left[u_{r}\right]≺\sigma_{\max}(\frac{1}{k_{\sigma }}+\frac{1}{\tilde{k_{\sigma }}})\;\\ \sigma^{\mathrm{int}}=0\;\;\;\;\;\left[u_{r}\right]≻\sigma_{\max}(\frac{1}{k_{\sigma }}+\frac{1}{\tilde{k_{\sigma }}})\; \end{array}\right\}$$

with $k_{\sigma }$ being the linear modulus, $\tilde{k_{\sigma }}$ the softening modulus, $\sigma_{max}$ the maximum bonding strength of the interface and $\left[u_{r}\right]$ the opening displacement between the particles and the matrix.

**Figure 6:**
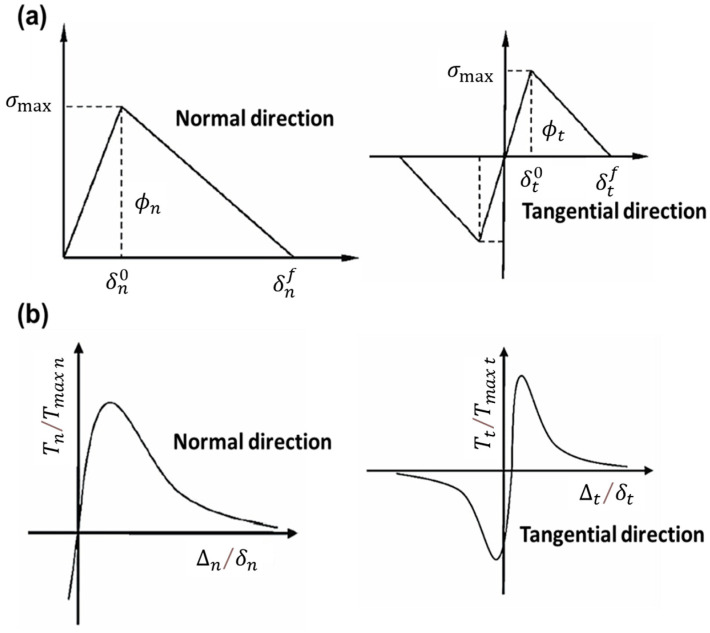
(**a**) Bilinear cohesion model; (**b**) exponential cohesion model [83].

The cohesive model characterizes the damage mechanical behavior of the interface between particles and the matrix of a composite solid propellant and idealizes the interface as a thickness-free surface with a certain bonding strength. The bonding performance at the interface directly affects the tensile strength of a solid propellant. This model was first proposed by Dugdale [80] and Barenblatt [81] to describe the damage evolution of the interface during material fracture. The bilinear cohesion model [82] had a simple structure and could well characterize mechanical behaviors such as damage evolution in the cohesive region under complex environments. The typical normal and tangential bilinear cohesion models are depicted in Figure 6a, and the governing equations [83] are Equations (13) and (14).

(13) $$T_{n}=\left\{\begin{array}{l} \frac{\sigma_{\max}}{\delta_{n}^{0}}\delta \;\;\;\delta \leq \delta_{n}^{0}\\ \sigma_{\max}\frac{\delta_{n}^{f}-\delta }{\delta_{n}^{f}-\delta_{n}^{0}}\;\;\;\delta ≻\delta_{n}^{0} \end{array}\right\}$$

(14) $$T_{t}=\left\{\begin{array}{l} \frac{\tau_{\max}}{\delta_{t}^{0}}\delta \;\;\;\delta \leq \delta_{t}^{0}\\ \tau_{\max}\frac{\delta_{t}^{f}-\delta }{\delta_{t}^{f}-\delta_{t}^{0}}\;\;\;\delta ≻\delta_{t}^{0} \end{array}\right\}$$

where $T_{n}\;and\;T_{t}$ are the normal and tangential stresses, $\sigma_{max}\;and\;\tau_{max}$ are the normal and tangential maximum stresses, $\delta_{n}^{0}\;and\;\delta_{t}^{0}$ are the critical opening displacements of the interface, and $\delta_{n}^{f}\;and\;\delta_{t}^{f}$ are the final cracking displacements. The critical fracture energies $\phi_{n}^{c}\;and\;\phi_{t}^{c}$ [83] are

(15) $$\left\{\begin{array}{l} \phi_{n}^{c}=\frac{1}{2}\sigma_{\max}\delta_{n}^{f}\\ \phi_{t}^{c}=\frac{1}{2}\tau_{\max}\delta_{t}^{f} \end{array}\right\}$$

The composite solid propellant will accelerate the damage under load. The bilinear cohesion model had limitations, and the nonlinear cohesion model was closer to the actual damage situation. Generally, the exponential cohesion model was used to describe the nonlinear mechanical properties of solid propellants. The stress of this model presented a nonlinear asymptotic zero in the process of decreasing, as depicted in Figure 6b. The fracture energy is as follows [83]:

(16) $$\phi (\Delta )=\phi_{n}+\phi_{n}\exp(-\frac{\Delta_{n}}{\delta_{n}})\left\{\left[1-r+\frac{\Delta_{n}}{\delta_{n}}\right]\frac{1-q}{r-1}-\left[q+(\frac{r-q}{r-1})\frac{\Delta_{n}}{\delta_{n}}\exp(-\frac{\Delta_{t}^{2}}{\delta_{t}^{2}})\right]\right\}$$

where ${∆}_{n}\;and\;{∆}_{t}$ are the normal and tangential displacements of the interface, $\delta_{n}\;and\;\delta_{t}$ are the critical opening displacement of the interface, $\phi_{n}$ is the normal cracking fracture energy, and q and r are

(17) $$q=\frac{\phi_{t}}{\phi_{n}}$$

(18) $$r=\frac{\Delta_{n}^{*}}{\delta_{n}}$$

where $\phi_{t}$ is the fracture energy of tangential cracking, and ${∆}_{n}^{*}$ is the normal displacement when tangential cracking is complete (normal stress is 0).

Xu et al. [84] combined the linear solid model and the exponential cohesion model to build a rate-dependent cohesion model and investigated the rate-dependent mechanical behavior of type “I” crack growth at the binder interface. Wang et al. [85] established a rate-dependent cohesion model based on the Kelvin model and the exponential cohesion model. Musto and Alfano [86] introduced internal damage variables to construct a linear viscoelastic genetic cohesion model. Chen et al. [87] introduced a rate-dependent damage function on the basis of the bilinear cohesion model and established a rate-dependent HTPB solid propellant interface type “II” cohesion model. However, mesoscopic mechanical parameters often need to be obtained by the inversion method based on macroscopic mechanical experiments, so accuracy cannot be guaranteed. Therefore, potential functions can be established on the basis of molecular dynamics [88] to calculate relevant parameters of the cohesion model at the microscale.

### 4.3. Mesoscopic Finite Element Model

As mentioned above, the analytical solution of mechanical properties of composite solid propellants can be obtained using the theory of mesoscopic mechanics, but the damage evolution mechanism under load is not involved, and the change mechanism of the internal mesostructure during damage cannot be discussed. Researchers consider combining mesomechanics theory with the finite element method, which can not only take the microstructure of a composite solid propellant into account, but also obtain calculation results with higher accuracy and greatly save calculation time and cost [89,90,91,92,93,94].

**Figure 7:**
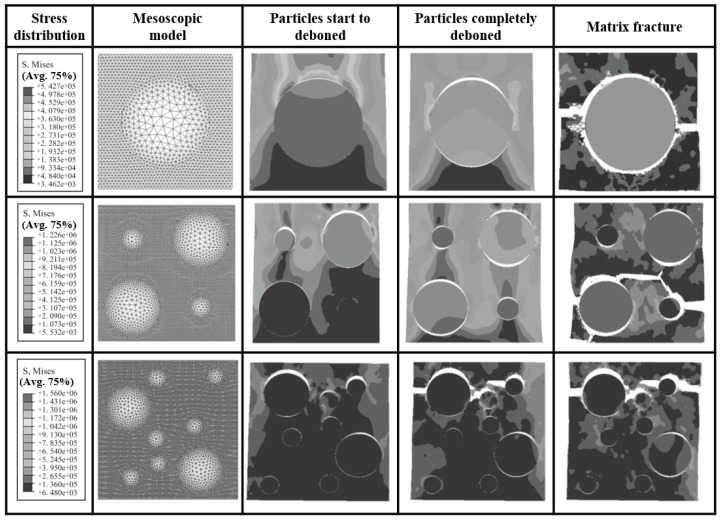
Damage and failure of single-particle, four-particle and eight-particle models under tensile action [99].

The particle-filled composite material was originally designed as a periodic distribution of single cells to realize the simplified mesostructure model of a composite solid propellant. Fang and Ning [95] investigated the effect of hexahedral and cuboid monocell arrangements on the effective modulus of particle-reinforced composites by numerical simulation. Peng et al. [96] established a viscoelastic finite element model of a composite solid propellant based on cylindrical cells and calculated and analyzed the stress distribution, damage location and form of the particle interface and matrix. Marur [97] calculated and analyzed the effective elastic modulus of particle-reinforced composites with a periodic distribution and the stress distribution of spherical particles under a uniaxial tensile load. Yuan et al. [98] established an axisymmetric unicellular model of a composite solid propellant and found that the stress field around the particles interacted. The larger the particles, the stronger the interaction, and the stress bands were easily formed, showing stress concentration. The smaller the particle, the smaller the interaction and the less obvious the stress concentration. Zhou et al. [99] established two-dimensional mesoscopic models of three solid propellants: a single particle, four particles and eight particles, and investigated the effects of particle number and size on the failure process of solid propellants through numerical simulation, as depicted in Figure 7. The results showed that the single-particle model was not sufficient to cause particle failure due to its high particle modulus. In the multi-particle model, the large particles were the first to deboned, and the matrix with a dense particle distribution was the first to break. The unicycle model and its periodic distribution model provided a simple method to analyze the mechanical properties of composite solid propellants and could determine the local stress–strain field inside the material. However, the simple and idealized element shape and particle distribution are not consistent with the actual situation, so they cannot be used for effective analysis.

**Figure 8:**
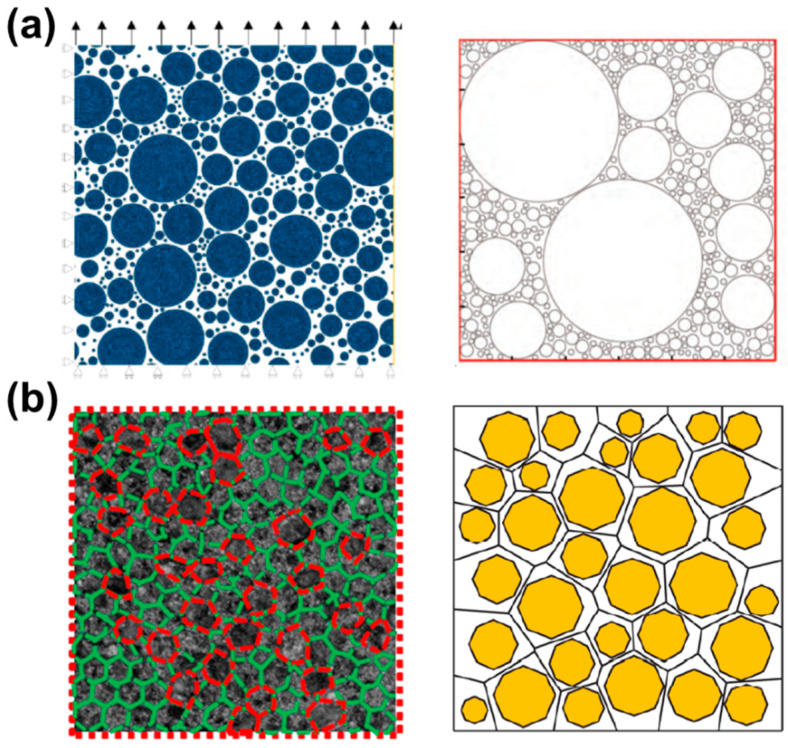
RVE models of composite solid propellant with (**a**) circular particles [100,102] and (**b**) polygonal particles [4].

The random algorithm can realize the particle-filling model of a composite solid propellant with different contents and particle size distributions, which is closer to the real mesoscopic structure. Most of the filled particles in composite solid propellants are spherical or nearly spherical particles. Some researchers [83,100,101,102] established RVE models of two-dimensional circular particle filling, as depicted in Figure 8a, to investigate the mechanical response behavior of composite solid propellants at the mesoscopic level. The numerical results showed that the material properties of the particles and the matrix were different, and interface debonding easily occurred near the large particles under the action of load, resulting in micropores. Then, the interface was further deboned; many micropores were formed in the dense particles, and the local stress of the matrix was concentrated near the large particles. As the load increased, the interface was completely deboned, forming microcracks that converged and produced macroscopic cracks until the composite solid propellant failed.

**Figure 9:**
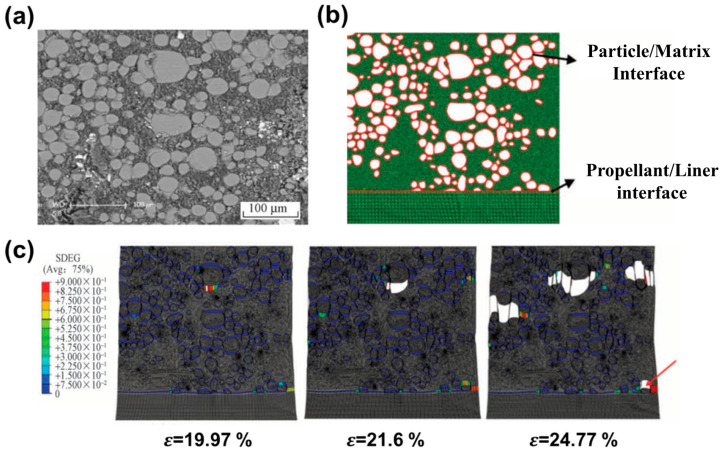
Composite solid propellant: (**a**) SEM topography; (**b**) finite element model; (**c**) SDEG cloud map with different strains (red arrow indicated that a particle deboned at the interface of the lining layer) [108].

Based on Voronoi elements, some researchers established an RVE model of polygon particle filling [4,103,104], as depicted in Figure 8b. Shen et al. [104] combined the Voronoi cell finite element method (VCFEM) and a homogenization method to obtain the variation rule of the equivalent mechanical properties of a composite solid propellant. Triangular elements and quadrilateral elements were used to divide each Voronoi element into integral regions. The mean stress and strain of the composite solid propellant mesoscopic model were as follows [105]:

(19) $$\left\{\begin{array}{l} {\bar{\sigma }}_{ij}=\sum_{m=1}^{N_{tri}}{\bar{\sigma }}_{m}^{tri}\frac{A_{m}^{tri}}{A_{RVE}}+\sum_{n=1}^{N_{quad}}{\bar{\sigma }}_{n}^{quad}\frac{A_{n}^{quad}}{A_{RVE}}\\ {\bar{\varepsilon }}_{ij}=\sum_{m=1}^{N_{tri}}{\bar{\varepsilon }}_{m}^{tri}\frac{A_{m}^{tri}}{A_{RVE}}+\sum_{n=1}^{N_{quad}}{\bar{\varepsilon }}_{n}^{quad}\frac{A_{n}^{quad}}{A_{RVE}} \end{array}\right\}$$

where $N_{tri}\;and\;N_{quad}$ are the numbers of triangle and quadrilateral regions, ${\bar{\sigma }}_{m}^{tri}\;and\;{\bar{\varepsilon }}_{m}^{tri}$ are the mean stress and mean strain of the triangle region, ${\bar{\sigma }}_{n}^{quad}\;and\;{\bar{\varepsilon }}_{n}^{quad}$ are the mean stress and mean strain of the quadrilateral region, $A_{m}^{tri}\;and\;A_{n}^{quad}$ are the areas of the mth triangle region and the nth quadrilateral region, and $A_{RVE}$ is the area of the RVE. The particle model of the random filling algorithm was relatively simple and had high randomness, and there was a certain difference from the real mesostructure of a composite solid propellant. Some researchers [106,107,108] used scanning electron microscopy (SEM), CT and other techniques to reconstruct the real micromorphology of a composite solid propellant. Zhang et al. [108] reconstructed the finite element model of a composite solid propellant based on SEM, as depicted in Figure 9b. The calculated results showed that under tensile load, damage evolution occurred on both the surface of large particles and the surface of the lining. However, the high cost of large instruments and the complexity of the model reconstruction algorithm limit the application of this method.

The mesoscopic constitutive model of a composite solid propellant is beneficial for analyzing the causes of damage and revealing the effect of mesoscopic structure evolution on mechanical properties. However, due to the large number of parameters in the mesoscopic model, it is difficult to obtain accurate data. The finite element method can be introduced to explore problems such as interface debonding, crack propagation, holes and aging at the mesoscopic level, but the authenticity of the model and the stability of the algorithm still need to be further improved.

## 5. Microscopic Molecular Model of Composite Solid Propellant

Molecular dynamics (MD) is a computational method combining force fields and classical mechanics and can be used to study the interaction between the components, structure and properties of materials at the molecular and atomic scales, and it has gradually become an effective means to study the macroscopic properties of composite solid propellants. COMPASS, Universal and other force fields were used to describe the interactions between molecules and atoms of each component of a composite solid propellant and to simulate the mechanical properties of single-component (such as HMX and RDX) [109,110], two-component [111] and multi-component systems.

The stress relaxation and creep of a composite solid propellant under load are related to the relative motion of the internal molecular structure. The above macroscopic constitutive model and mesoscopic mechanical model do not involve the multi-component structure and the interaction between each component and particles, and they cannot reveal the failure mechanism of matrix damage and fracture, particle dehumidification and fragmentation. With the improvement of computer performance and the development of mechanics theory, the molecular dynamics method plays an important role in the development of composite solid propellants.

Lubachevsky and Stillinger [112] first applied the molecular dynamics algorithm to the particle-filling problem of the solid phase. Later, Kansal et al. [113] established the particle-filling model of high solid-phase content on this basis, and Knott et al. [101] adopted this algorithm to generate a particle-filling model of a homogeneous composite solid propellant. Kochevets [114] considered parallel optimization to generate a three-dimensional structure model of a composite solid propellant and investigated the structure law of a propellant with a graded particle size distribution, which greatly improved the computational efficiency. Ghossein and Lévesque [115] proposed an effective collision time algorithm to detect whether there was an overlap between particles and generate ellipsoidal particles. Stafford and Jackson [116] modified the molecular dynamics algorithm to generate a filling model of irregularly shaped solid particles, such as cylindric and polyhedral particles.

Yu et al. [117] used molecular dynamics method to investigate the binding energy and mechanical properties of four systems and found that the binding energy of (PEG/NG/BTTN)/AlH3 system was the highest, and the strong polar AlH3 crystal had the highest binding energy with polyhydroxy PEG molecules. The radial distribution function showed that the O atom in PEG had a strong hydrogen bond with the Al atom in AlH3. Xia et al. [118] simulated the mechanical properties of NG and NG/TEGDN systems at low temperature and found that TEGDN significantly reduced the rigidity of NG and improved the ductility, so the mechanical properties of the mixed plasticizer were better. Lan et al. [119] established a physical mixing model of PET/N-100 to simulate the crosslinking process of the binder matrix, which can predict the macroscopic properties. Fu et al. [120,121] calculated the mechanical properties of NEPE composite solid propellant and the interface binding energy of PEG/Al using molecular dynamics and explained the “dewetting” mechanism between the solid particles and binder matrix. Qu et al. [122] calculated the interaction between CL-20 and NG, and the results showed that N-C bonds and N=O bonds of CL-20 and O-N bonds, N=O bonds and C-C bonds of NG were all smaller than the bond lengths of elemental materials, indicating that the CL-20/NG mixture had good stability and bonds that were not easy to break.

Zhou and Lu [123] established the evolution equation of viscoelastic deformation of materials based on the theory of molecular kinematics and derived the stress–strain relationship of integral linear viscoelastic materials combined with the basic equation of thermodynamics:

(20) $$\sigma_{ij}=\int_{-\infty }^{t}E_{ijkl}(t-\tau )\frac{\partial \varepsilon_{kl}(\tau )}{\partial \tau }d\tau +F_{ij}(t-\tau )\frac{\partial \theta (\tau )}{\partial \tau }d\tau$$

This equation combined the macroscopic mechanical parameters of viscoelastic materials with the microscopic molecular motion parameters and can explain the constitutive relation of integral viscoelastic materials from the microscopic molecular level.

The simulation and prediction of mechanical properties of composite solid propellants using molecular dynamics algorithms can reveal the relationship and mechanism between the microscopic molecular structure and macroscopic mechanical properties and provide a theoretical basis and reference for component design, structural optimization and performance improvement. However, due to the development of micromechanics and the limitation of computer levels, it is very difficult to establish and calculate large-scale molecular models. For composite solid propellants containing more components, it is almost impossible to establish more accurate molecular models and accurate force fields, and the development is not yet mature. Therefore, the investigation of composite solid propellants at the microscopic molecular and atomic scales still faces many challenges, and the previous studies are not sufficient, requiring more in-depth exploration and analysis.

## 6. Conclusions and Prospect

At present, a great deal of work has been conducted on the characterization of mechanical properties, the establishment of macroscopic constitutive models, mesoscopic mechanical models and microscopic molecular models, and the optimization design of composite solid propellants. This paper reviewed the numerical simulation of composite solid propellants based on model scale and modeling methods, followed the historical progress of key models, summarized the main achievements in this field, and emphasized the importance of the establishment of numerical calculation models in the investigation of mechanical properties and structural optimization design. However, there are still many key problems to be solved:

1. Although a macroscopic constitutive model can describe the nonlinear stress–strain relationship of a composite solid propellant well, it cannot explain the mechanism of microstructure change during the loading condition.
2. A macroscopic constitutive model with additional particle structure parameters (particle size, shape) and high strain rate (greater than 10^3^ s^−1^) needs further investigation.
3. Mesoscopic mechanical models can explain the mechanism of the microstructure evolution of composite solid propellants, but most of the models are based on various algorithms and describe the local microstructure of composite solid propellants, which is greatly affected by random factors.
4. Further research is needed on how to construct a real and effective mesoscopic structure model of composite solid propellants, ensure the stability of the algorithm and reduce the cost.
5. The deformation mode and failure mechanism of composite solid propellants under explosion load and ultra-high strain rate, as well as the design structure and performance optimization of additive manufacturing [124,125,126,127,128,129], are the key points for the future research of composite solid propellants.

**Figure 10:**
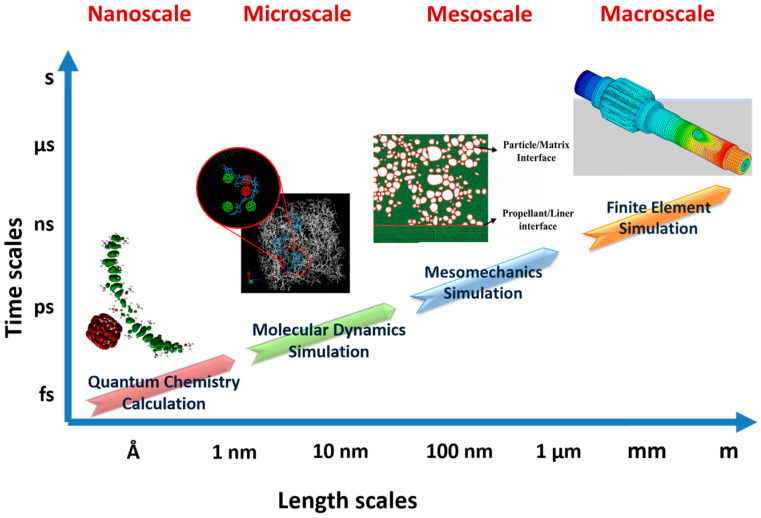
Nano-micro-meso-macroscale simulation method.

Composite solid propellants are developing rapidly. With the development and improvement, the design of the composite solid propellant ratio, structure optimization and performance improvement will face greater challenges:

1. The constitutive model of a composite solid propellant needs to be suitable for various complex loads.
2. Based on the molecular ratio of the material, the precise parameters of all aspects of the structure are determined, and the nano- micro-, meso- and macroscale calculation of the composite solid propellant needs to be developed, as in Figure 10.

3. There is a need to improve additive manufacturing technology, develop excellent formulas, optimize process parameters and scale up the process, and realize integrated printing of solid propellants.

## Figures and Tables

**Table 1:** Performance comparison of various models.

Model	Features	Weaknesses
Macroscopic constitutive model	Can describe nonlinear viscoelastic mechanical properties; Ignores the microstructure.	♦ Cannot explain the mechanism of the damage failure process; ♦ Only considers particle content and strain rate, regardless of the particle size and shape; ♦ Cannot describe the mechanical behavior under large strain and high strain rate.
Mesoscopic mechanical model	Considered as a composite with matrix and particles; Can quickly calculate the stress, strain and stiffness; Combines mesoscopic mechanics and finite element method to describe the deformation mechanism.	♦ The simple shape and regular permutation are too idealistic and far from the realistic structure; ♦ Cannot guarantee the authenticity of model, the accuracy of parameters and the stability of the algorithm.
Microscopic molecular model	Reveals the relationship and mechanism between microscopic molecular structure and macroscopic mechanical properties at molecular and atomic scales.	♦ The establishment and calculation of large-scale molecular models are relatively difficult; ♦ Unable to establish an accurate microscopic molecular model and accurate force field.

## Data Availability

The data used during the study are available from the first author and corresponding author by request.
